# The association between early in marriage fertility pressure from in-laws’ and family planning behaviors, among married adolescent girls in Bihar and Uttar Pradesh, India

**DOI:** 10.1186/s12978-021-01116-9

**Published:** 2021-03-09

**Authors:** Anvita Dixit, Nandita Bhan, Tarik Benmarhnia, Elizabeth Reed, Susan M. Kiene, Jay Silverman, Anita Raj

**Affiliations:** 1 Center on Gender Equity and Health, Division of Infectious Diseases and Global Public Health, School of Medicine, University of California San Diego, 9500 Gilman Drive #0507, La Jolla, CA 92093-0507 USA; 2 Joint Doctoral Program in Public Health, San Diego State University-University of California San Diego, 9500 Gilman Dr, La Jolla, CA 92093 USA; 3 Scripps Institution of Oceanography, University of California San Diego, 8622 Kennel Way, La Jolla, CA 92037 USA; 4 Department of Family Medicine and Public Health, University of California San Diego, 9500 Gilman Dr, La Jolla, CA 92093 USA; 5 Division of Health Promotion and Behavioral Science, School of Public Health, San Diego State University, 5500 Campanile Dr, San Diego, CA 92182 USA; 6 Division of Epidemiology and Biostatistics, School of Public Health, San Diego State University, 5500 Campanile Dr, San Diego, CA 92182 USA; 7 Department of Education Studies, Division of Social Sciences, University of California San Diego, 9500 Gilman Dr, La Jolla, CA 92093 USA

**Keywords:** In-laws’ pressure, Married adolescent girls, Fertility, Family planning, India

## Abstract

**Background:**

Married adolescent girls are vulnerable to risky sexual and reproductive health outcomes. We examined the association of fertility pressure from in-laws’ early in marriage with contraceptive use ever, parity, time until first birth, and couple communication about family size, among married adolescent girls.

**Methods:**

Data were taken from a cross-sectional survey with married girls aged 15–19 years (N = 4893) collected from September 2015 to July 2016 in Bihar and Uttar Pradesh, India. Multivariable regression assessed associations between in-laws’ fertility pressure and each outcome, adjusting for sociodemographic covariates.

**Results:**

We found that 1 in 5 girls experienced pressure from in-laws’ to have a child immediately after marriage. In-laws’ fertility pressure was associated with lower parity (Adj. β Coef. − 0.10, 95% CI − 0.17, − 0.37) and couple communication about family size (AOR = 1.77, 95% CI 1.39, 2.26), but not contraceptive use or time until birth.

**Conclusions:**

Our study adds to the literature identifying that in-laws’ pressure on fertility is common, affects couple communication about family size, and may be more likely for those yet to have a child, but may have little effect impeding contraceptive use in a context where such use is not normative.

**Supplementary Information:**

The online version contains supplementary material available at 10.1186/s12978-021-01116-9.

## Plain English summary

Adolescent girls who marry early are vulnerable to poor reproductive health outcomes including low contraception use and unwanted pregnancy due to low decision-making agency and communication with their husbands. Married adolescent girls in India live in a gender inequitable context facing fertility pressures from their in-laws and extended family rooted in social norms. This study used survey data from married girls aged 15–19 years in India, to study the association of in-laws' pressure to have a child immediately after marriage with contraceptive use ever, parity, time until first birth, and couple communication about family size. We found that experience of in-laws' fertility pressure was common, and was associated with lower parity and couple communication about family size, but not contraceptive use or time until birth. Adolescent Reproductive and Sexual Health (ARSH) programs should include more focus on raising adolescent consciousness regarding contraceptive use and delayed first birth as potential choices in marriage, social norms related to unacceptability of fertility pressures from in-laws’ and delayed first birth in marriage.

## Background

Contraceptive use and family planning reduce unplanned pregnancy and prevent maternal and newborn morbidity and mortality [1], and may be particularly important for adolescent girls. Globally among girls aged 15–19, one in six is married and about 16 million give birth annually [2, 3]. They often lack knowledge, agency, and resources to make family planning decisions [4]. Their agency, specifically their decision-making ability, is a key driver of family planning and fertility behaviors like contraceptive use [5]. However, the issue is complex in India due to strong patrilocal (married couples living with or near husbands’ parents) and patrilineal (defining descent solely through the father/male line) practices. Women and girls often lack control over family planning and fertility decisions such as timing of pregnancy, family size, and contraception due to extended family’s influence [6, 7]. Fertility pressures from in-laws’ may be a particular concern, especially for adolescent wives who are more likely to be in joint families or residing near in-laws’ [8].

India is committed to increasing modern contraceptive use, and increasing female age at first birth while prioritizing adolescent health policy [9–11]. However social norms related to family planning and gender equality (e.g. early marriage, son-preference, pro-fertility norms, and toxic masculine ideology leading to violence and reproductive coercion by husbands and in-laws) continue to hold back progress on these issues [12, 13]. The states of Bihar and Uttar Pradesh (UP) have been grappling with large populations (99 million and 200 million respectively) with fertility rates (3.4 and 2.7 per woman respectively) significantly greater than the national average (2.2 per woman). The contraceptive use has fallen in the last decade from 41.3% to 32.1% in Bihar, 39.8 to 42.4% in UP, compared to 55.8% to 51.5% nationally. Child marriage among girls is 41.9% in Bihar, 22.9% in UP, and 27.9% nationally at 18–29 years. The most vulnerable are adolescent girls who live in rural areas, who have lower educational attainment, and who marry young, which leads to a myriad of poor outcomes [8]. These state contexts allow us to understand pressures of a gender inequitable ecosystem on vulnerable married adolescents and their fertility outcomes. There is growing evidence on social norms and practices showing that pronatalism (socially desirable pro-birth norms), pregnancy early in marriage, and fertility decision determined by husband and in-laws limit women’s agency to practice beneficial family planning and fertility behaviors [7, 14, 15]. These practices are rooted in gender-based power, where male members (usually husbands) followed often by mother in-laws’ (as the husbands family), often have decision-making control over family planning and fertility that are family-decisions and not nuclear (i.e. husband and wife) decisions [16, 17]. In such contexts, adolescent girls may have limited or no power over their reproductive health including use of family planning services [18].

Assessments of female family planning and fertility behaviors and experience of coercion have largely focused on girls’ parental characteristics, such as mother’s education, parental wealth, husband's influence or women’s household decision-making, but lack understanding of in-laws’ influence. However, pressure from in-laws’ is conceptually complex since in-laws’ are likely to have an influence both directly on the girl and indirectly through husbands, and this in-laws’ influence is associated with a higher likelihood of larger desired family size if a couple is living in an extended family [19].

Study of in-laws’ pressure has been largely limited to qualitative examination of in-laws’ influence over fertility decisions and the association with family planning and fertility outcomes has not been well established. No previous surveys quantified how in-laws’ pressure to have a child immediately after marriage influences family planning and fertility behaviors among adolescent girls, which is important because girls may be most sensitive and vulnerable to such pressures immediately after marriage. Further, assessments have not looked at whether women report their perception of feeling in-laws’ pressure to have a child immediately after marriage, or whether in-laws’ pressure may influence couple communication on family size and actual number of children. It is well established that women’s decision-making, couple communication, and agreement on contraception and fertility leads to increased contraceptive use [20, 21]. Even though couple communication is associated with contraceptive use, research has not examined whether in-laws’ fertility pressure affects this communication. Moreover, it is crucial to conceptualize and study in-laws’ pressure because the evidence on consequences of in-laws’ control over women’s fertility extends to extreme forms such as intimate partner violence and reproductive coercion behaviors of husbands and in-laws that interfere with women’s fertility decisions [22–24].

We assessed the association of early in marriage fertility pressure from in-laws and the following contraceptive and fertility behaviors: contraceptive use ever, parity, time until birth, and couple communication about family size among a sample of married adolescent girls age 15–19 years in the states of Bihar and UP (UP in India. Findings from this study may inform public healthcare guidelines and policies to include in-laws’ in family planning intervention programming to reduce the risk of undesirable fertility outcomes, especially for high-needs populations in India and similar country contexts.

## Methods

### Study design

We analyzed data from a cross-sectional survey of 5206 married adolescent girls age 15–19 years from the “Understanding lives of adolescents and young adults” (UDAYA) study conducted from September 2015 to January 2016 in Uttar Pradesh (N = 1798) and January to July 2016 in Bihar (N = 3408). A stratified multistage systematic sampling for rural and urban sampling units was used in both states, from which systematic sampling for boys and girls of specific age categories (boys 10–14, girls 10–14, boys 15–19, girls 15–19, married girls 15–19) was carried out to yield the desired survey sample size providing state representative estimates using weighted data [25].

### Data collection

Trained field research investigators conducted in-person interviews of adolescents with parents/guardians’ consent. Self-report data were collected on socio-demographics, media exposure, parental interaction/relationship, communication, mobility and decision-making, gender and self-efficacy, sexual reproductive matters, connectedness and friendship, marriage process and life, sexual experiences, health-seeking, substance use and violence, political participation, and biomarkers. Data quality and fieldwork were monitored by trained field coordinators and Population Council research staff.

### Measures

The dependent variables were (a1) contraceptive use ever established from the survey question “Have you/your husband ever used any method to prevent or delay pregnancy?” with response categories Yes, No, (a2) ever modern contraception use was calculated from “Which method(s) did you/he use?” and categorized as none, traditional (rhythm, withdrawal, and other), and modern (pill, IUD [Intrauterine Device], injectables, implants, condom, diaphragm, foam/jelly, female sterilization, male sterilization, female condom, and LAM [Lactational Amenorrhea Method]), (b) parity from the question “Have you ever given birth to a live child? If yes, how many live births?”, with continuous responses ranging from 0 to 3 (2 girls who reported having had 4 children were also marked as 3 children), (c) time until birth from difference between ‘Age at first birth’ and ‘Age at marriage’, with continuous responses ranging from 0 to 7 years, (d) couple communication about number of children from “Did you and your husband ever discuss about how many children to have before the first time you became pregnant?”, with response categories Yes, No/Don’t remember.

The independent variable of in-laws’ pressure to have a child immediately after marriage was measured from the question “Did your in-laws’ or other family members pressure you to have a child immediately after marriage?”, with response categories Yes, No.

We included confounding variables including age (continuous, range 15–19 years), education (continuous, range 0–15), residence (Urban, Rural), religion (Hindu, Non-Hindu), caste (General, Scheduled caste/tribe, Other backward castes, Other/Don’t know), time since marriage (continuous, range 0–11 years), wealth index quintile as a marker of household socioeconomic status, and state (UP, Bihar). Wealth quintile was calculated by assigning weights to household assets ranking households (ranging from 0–57) which were then categorized into quintiles from poorest (Q1) to wealthiest (Q5). Example household assets measured in the survey are durable goods such as television, means of transport and amenities such as cooking fuel, drinking water and electricity [25, 26].

For descriptive purposes we looked at whether girls reported a fear of being called barren using the survey question; “Were you afraid that your in-laws and others would call you barren if you didn’t have a child soon after you got married?”, with response categories Yes, No.

### Statistical analysis

Our inference focused on married girls so analytic dataset excluded girls who were currently not married or cohabiting with their husbands. Survey specific weights were used for all analysis to ensure state representative estimates [25]. One-way and two-way descriptive frequencies and weighted proportions were calculated for the independent variable with dependent variables. Logistic and linear multivariable regressions were used to model the relationship between in-laws’ pressure to have a child immediately after marriage with (a1) ever contraception use, (a2) ever modern contraceptive use as a multinomial regression sensitivity analysis, (b) parity (c) time until birth, and (d) couple communication about number of children, adjusting for all potential sociodemographic confounders listed above, which were chosen a priori based on literature and author expertise [27–29]. Further, state stratified exploratory analysis was done in recognition of differences in health systems in the two states (see Additional file 1: Appendix). No multicollinearity was found between confounders using a Variance Inflation Factor (VIF) cutoff of 4 [30]. All analyses were conducted using STATA 14.0 [31].

## Results

### Sociodemographic characteristics

Table 1 presents the sociodemographic characteristics of adolescent girls in the study. The adolescent girls were aged 15 to 19 years, with a majority being 18 and 19 years old (3622 of total 4893 sample). They had a mean education of 6.31 years (SD 0.14), majorly resided in rural areas (3013), identified as Hindu (4097), and belonged to marginalized castes including Scheduled Caste, Scheduled Tribe, and Other Backward Castes (4385). Their wealth quintiles were distributed through Q1 poorest (806), Q2 poorer (937), Q3 middle (1154), Q4 richer (1233), and Q5 richest (765).

**Table 1:** Sociodemographic characteristics of married adolescent girls (15–19 years) in Bihar and Uttar Pradesh, India (N = 4893)

	Total		
	Overall N (%)	In-laws’ pressure	
		Yes, n (%)	No, n (%)
*Age (years)*			
15	117 (1.83%)	20 (1.93%)	97 (1.81%)
16	369 (5.55%)	81 (8.15%)	288 (6.18%)
17	785 (15.26%)	174 (17.25%)	611 (14.81%)
18	1610 (32.55%)	323 (32.45%)	1287 (32.57%)
19	2012 (43.82%)	355 (40.22%)	1657 (44.63%)
Education (years), mean (SD)	6.31 (0.14)	6.08 (4.68)	6.36 (4.30)
*Area of residence*			
Rural	3013 (85.31%)	622 (88.11%)	2391 (84.68%)
Urban	1880 (14.69%)	331 (11.89%)	1549 (15.32%)
*Religion*			
Hindu	4097 (81.66%)	826 (85.29%)	3271 (80.84%)
Other religions*	796 (18.34%)	127 (14.71%)	669 (19.16%)
*Caste*			
General	508 (12.98%)	90 (10.11%)	418 (13.63%)
SC/ST/OBC**	4385 (87.02%)	863 (89.89%)	3522 (86.37%)
Time since marriage (years), mean (SD)	2.18 (1.41)	2.29 (1.50)	2.14 (1.38)
*Wealth quintile*			
Q1 (poorest)	806 (14.56%)	170 (16.79%)	636 (14.05%)
Q2 (poorer)	937 (20.15%)	205 (20.26%)	732 (20.12%)
Q3 (middle)	1154 (23.36%)	233 (26.88%)	919 (22.56%)
Q4 (richer)	1233 (24.20%)	234 (22.61%)	999 (24.56%)
Q5 (richest)	765 (17.73%)	111 (13.45%)	654 (18.70%)
Total N	4893 (100%)	953 (100%)	3940 (100%))

Frequency and weighted proportions are reported for categorical variables. Weighted means and standard deviations are reported for continuous variables

^*^Other religions include Muslim, Christian, Buddhist, and Others

^**^SC: Scheduled Caste, ST: Scheduled Tribe, OBC: Other Backward Caste

### Experience of pressure from in-laws’ to have a child and family planning and fertility outcomes

Nearly one in five (18.45%) married adolescent girls report experiencing pressure from in-laws or other family members to have a child immediately after marriage, while 81.55% did not report pressure. In this sample, 18.83% girls also reported that they were afraid their in-laws’ would call them barren if they didn’t have a child soon after marriage (Additional file 1: Appendix Table A5). Among those who reported pressure from in-laws to have a child immediately after marriage, 12.63% reported ever use of any contraception (with overall 8.45% using a modern method), while 15.89% of those who did not report in-laws’ pressure report using contraception. Among those who reported in-laws’ pressure, 87.37% were non-users of contraception, and among those who did not report pressure, 84.11% were non-users of contraception. Further, 66.79% of those who reported in-laws’ pressure reported ever communication with husband about number of children, while 44.79% of those who did not report in-laws’ pressure reported that they have had the communication. Among those who reported in-laws’ pressure, 33.21% said did not or don’t know to having had the communication, while among those who did not report in-laws’ pressure 55.21% reported no or don’t know to having had the communication. The average time from marriage until birth was 1.70 years (SD 1.09) among those who reported in-laws’ pressure, and 1.62 years (SD 1.00) among those who did not report in-laws’ pressure. The average parity was 0.42 (SD 0.64) among those who reported in-laws’ pressure, and 0.47 (SD 0.64) among those who did not report in-laws’ pressure (Table 2). We also conducted an exploratory state-wise analysis (Additional file 1: Appendix Tables A1, A2, A3).

**Table 2:** Pressure from in-laws to have child early in marriage by outcomes of ever contraception use, communication about number of children, time until first birth, and parity, among married adolescent girls (15–19 years) in Bihar and Uttar Pradesh, India (N = 4893, and N = 2202 for communication about number of children)

	Overall, n (%)	Ever contraception use, n (%)		Communication about number of children, n (%)		Time until first birth (0–7 years), mean (SD)	Parity (range 0–4 births), mean (SD)
Pressure from in-laws’	-	Yes	No	Yes	No/Don’t know		
Total							
Yes	953 (18.45%)	115 (12.63%)	838 (87.37%)	612 (66.79%)	341 (33.21%)	1.70 (1.09)	0.42 (0.64)
No	3940 (81.55%)	586 (15.89%)	3354 (84.11%)	1787 (44.79%)	2153 (55.21%)	1.62 (1.00)	0.47 (0.64)
Total N	4893 (100%)	701 (15.29%)	4192 (84.71%)	2765 (57.34%)	2128 (42.66%)	2202	4893

Frequency and weighted proportions are reported for categorical variables. Weighted means and standard deviations are reported for continuous variables

### Association between pressure from in-laws’ to have a child and family planning and fertility outcomes

Multivariable analysis indicates that those who report in-laws’ pressure to have a child immediately after marriage are more likely to report having discussed with their husbands how many children to have before first pregnancy (AOR = 1.77, CI = 1.39–2.26), and to have lower parity (Adj. β Coef. − 0.10, 95% CI − 0.17, − 0.37), after adjusting for confounders (Table 3). In-laws’ pressure was not associated with ever use of contraception (or modern contraceptive use ever, see Additional file 1: Appendix Table A4) or with time until birth.

**Table 3:** Unadjusted and adjusted logistic and linear regression between pressure from in-laws’ to have child early in marriage by outcomes of ever contraception use, communication about number of children, time until birth, and parity, among married adolescent girls (15–19 years) in Bihar and Uttar Pradesh, India (N = 4893)

	Ever use of contraception		Communication about number of children		Time until birth (N = 2202)		Parity	
In-laws’ pressure to have children	Unadjusted	Adjusted	Unadjusted	Adjusted	Unadjusted	Adjusted	Unadjusted	Adjusted
	OR (95% CI)	AOR (95% CI)	OR (95% CI)	AOR (95% CI)	β Coef. (95% CI)	β Coef. (95% CI)	β Coef. (95% CI)	β Coef. (95% CI)
No	Ref	Ref	Ref	Ref	Ref	Ref	Ref	Ref
Yes	0.76 (0.53, 1.11)	0.83 (0.58, 1.20)	**1.64 (1.28, 2.08)**	**1.77 (1.39, 2.26)**	0.08 (− 0.09, 0.25)	− 0.04 (− 0.19, 0.12)	− 0.05 (− 0.12, 0.01)	− **0.10 (**− **0.17, **− **0.37)**

Adjusted for age, education, residence, religion, caste, time since marriage, wealth quintile (combined). *OR* odds ratio, *CI* confidence interval, *ref* reference. ORs and AORs are show for logistic regressions. β Coef. and adjusted β Coef. are shown for linear regressions. ORs in bold represent *p* < 0.05

A sensitivity analysis to assess the association of in-laws’ pressure to have a child immediately after marriage with parity was carried out only among girls who reported at least 1 birth. The estimate continued to be in the same direction, but association decreased in this sample (Adj. β Coef. − 0.04, 95% CI − 0.10, − 0.02).

## Discussion

We found that in-laws’ pressure to have a child immediately after marriage is a common experience in our sample of married adolescent girls with almost one in five girls reporting it. This notion of in-laws’ pressure has been found in other studies among women that show mother-in-laws’ influence on family planning and fertility decisions [7, 32, 33]. Besides, girls feel pressure to prove their fertility early in marriage in this context, since they also reported a fear of being called barren due to lack of a child, although with a caveat that it may be associated with time since marriage. This fear may stem from a context where girls may be stigmatized for not having a child or are worried about not being able to secure their position in the household early by having a child, and experience stress from fertility pressures despite their young age. Previous assessment of attitudes has found that women may be blamed for not having children soon after marriage, which may be considered a sign of infertility or marital happiness [34]. In another similar context women have reported psychosocial health problems including anxiety, stress and depression at the psychological level and social isolation and stigma at the social level due to an inability to get pregnant [35]. Thus, in-laws’ pressure may need to be considered when examining girls’ family planning and fertility behaviors, and not just restricted to husbands' influence.

In-laws’ pressure to have a child immediately after marriage was associated with couple communication about number of children before first pregnancy, after adjusting for socio-demographics and time since marriage. Although, the outcome of higher communication between spouses may seem to be positive or programmatically desirable for family planning interventions, we cannot confirm that it is desired in this population. Report of communication between girls and their husbands may suggest increased female reproductive agency, however, we do not know the nature of their reported communication and whether it is by choice. Spousal communication needs further scrutiny since couples’ decision-making concordance and quality of relationship have a direct bearing on contraceptive use [20, 36], especially for married adolescent girls with limited agency in a gender unfriendly context. These reports of communication could have been due to in-laws’ pressure, conflicts with husbands, or pressure from husbands. We also do not know about recent or ongoing pressure from in-laws, since we only measured pressure experienced immediately after marriage.

There was an association of in-laws’ pressure with lower parity, after adjusting for socio-demographics and time since marriage. Our counter-intuitive findings may be a back effect or reverse causation of girls experiencing in-laws’ pressure in response to an absence of or lower parity which cannot be established temporally from retrospectively collected cross-sectional information. Longitudinal study could explore whether this finding is indicative of girls experiencing pressure from in-laws due to delay in having children. Also, the same association of in-laws’ pressure with parity did not sustain in a sensitivity analysis among girls who reported at least one birth. This sensitivity analysis was done in recognition of the young age of girls in our sample meaning girls may not have had any children yet and may be far from meeting their fertility goals. The estimate of the association of in-laws' pressure to have a child immediately after marriage with lower parity was decreased and approaching non-significance, among girls who reported at least one birth. This suggests that in-laws’ pressure is experienced by girls with 0 or low births. Lower parity in adolescence may be attributed to a lower likelihood of pregnancy due to irregular periods especially among girls with compromised nutritional status in Low-to Middle-Income Countries (LMICs) [37, 38].

In-laws’ pressure to have a child immediately after marriage was not associated with ever use of contraception and time until birth. We did not see an association with contraception use, perhaps due to a low prevalence of contraception use in this young sample of adolescent girls who may still be far from meeting their fertility goals. Moreover, women in India do not use contraceptives until desired parity and sex composition is achieved, after which a majority opt for permanent contraceptives [8, 39] so adolescent girls may not be using contraception yet as seen from the 15.29% use of contraceptives reported in this sample and 15.89% use among girls not reporting in-laws’ pressure, which is low. These girls may not want to delay their first birth due to pressure felt from these pronatal social norms and fear of not meeting expectations. Family planning outcomes may not be on the radar for these young adolescents yet. Previous studies have found that mother-in-laws’ desire for number of grandchildren is associated with their daughter-in-laws’ preferred family size [40], and mother-in-laws’ influence is associated with daughter-in-laws’ reporting a low likelihood of visiting a family planning clinic and use of modern contraceptives [33]. Perhaps husbands are more involved in contraception, but the extended family only exerts pressure on fertility. So these findings add complexity to the literature on other measures of in-laws’ control over family planning and fertility such as desired parity measured in comparison with mother-in-laws [18, 32, 40], and direct coercion or interference of in-laws in women’s family planning access, initiation, or continuation [23]. However, these findings do not clarify how it affects adolescent wives. Furthermore, contradictory findings to what previous national analysis reported [41], have shown that living with mother-in-laws can result in increased use of modern contraceptives and institutional delivery among women and girls aged 15–49 years [42], perhaps due to increased social and financial support from them but needs further clarification. However, these data are also cross-sectional, thus suggesting further need for longitudinal studies to assure the direction of causality.

Findings of this study should be interpreted keeping in mind its limitations with the key limitation of the cross-sectional design resulting in a possible back-effect. To minimize bias of a chronological timeline back-effect, we adjusted the multivariable models for time since marriage. Although longitudinal data on adolescents' sexual and reproductive health is warranted, recent cross-sectional data gives the most up to date picture of current dynamics to inform policy, given that the indicators and predictors are rapidly changing. Moreover, the adolescent girls reporting of in-laws' pressure was retrospective and subject to recall bias. It was also non-specific, so we were unable to understand when they were pressured. Further research on improved measurement is needed since literature has not focused on measures of recent or ongoing fertility pressure from in-laws. Qualitative studies can help disentangling the kinds of pressures experienced and consequences of these pressures on fertility decision-making and behavior. Further, there may be other markers of access to family planning services beyond intra-family relationships that need distinctive examination to understand use of family planning per se, especially to understand lack of the associations that were expected in this analysis. For example, living with in-laws or joint family has been noted as an impactful variable in previous analyses [33, 42], but our study data was lacking such a measure on co-habitation with in-laws. Another limitation of the outcome on parity is that it does not include miscarriages, stillbirths, and abortion. Further study is needed to explain reasons behind in-laws’ pressure since girls in the current sample also reported that they were afraid their in-laws would call them barren if they didn’t have a child soon after marriage. There is a need to understand girls’ and couples’ fertility goals with recognition that infertility concerns may need to be addressed beyond just family planning. Moreover, pressure from in-laws may be due to son-preference norms in India. Future qualitative work needs to explore son preference and whether it is changing.

## Conclusions

To conclude, findings show that in-laws’ pressure to have a child immediately after marriage is prevalent in these vulnerable contexts. Married adolescent girls who experience such in-laws’ pressure are more likely to report communication with their husbands on family size, and a lower parity, but we did not observe associations with having ever used contraceptives and delay/time until first birth. This gender and power-based family dynamic of in-laws’ pressure needs to be accounted for when considering family planning and fertility decision-making. Currently, in-laws or extended family is not included in counseling in family planning programs. Not addressing in-laws’ pressure as a form of in-laws’ involvement in fertility decision-making may impede the goals of providing person-centered and gender-equitable care [43]. Considerations of coercion have been effective in interventions [44, 45], which given our findings suggests that there may be value in further research on in-laws’ pressure that could inform an intervention approach to pressure broadly. If in-laws prevent girls’ agency and continue to be the decision-makers around family planning and fertility, given the belief that these decisions affect the entire household, then there is a need to include them in the family planning conversation during the provision of healthcare services. This is crucial for countries with patrilocal societies like India where living in extended households is common. However, when future research simplifies the complexity in the value of in-laws’ pressure for fertility outcomes, any intervention development must be context-specific and carefully designed to be rooted in improving girls’ agency as the entry point, so as not to reinforce in-laws’ power as decision-makers. This study shows that in-laws’ pressure is an important issue for married adolescent girls given its prevalence and associations with communication on number of children and parity. However, there is a need for future research to study the causal direction between in-laws’ pressure, couple communication, and parity to effectively include in-laws and family in programs.

## Supplementary Information

**Additional file 1: Table A1.** Sociodemographic characteristics of the study sample among married adolescent girls (15–19 years) in Bihar and Uttar Pradesh, India (Bihar N = 3,182, UP N = 1711). **Table A2.** Pressure from in-laws’ to have child early in marriage by outcomes of ever contraception use, communication about number of children, time until first birth, and parity, among married adolescent girls (15–19 years) in Bihar and Uttar Pradesh state wise, India (Bihar N = 3182, and Uttar Pradesh N = 1711, 2202 for communication about number of children). **Table A3.** Unadjusted and adjusted logistic and linear regression between pressure from in-laws’ to have a child immediately after marriage by outcomes of ever contraception use, communication about number of children, time to birth, and parity, among married adolescent girls (15–19 years) in Bihar (N = 3182), and Uttar Pradesh (N = 1711), India. **Table A4.** Sensitivity analysis of unadjusted and adjusted multinomial regression between pressure from in-laws’ to have child early in marriage and ever modern contraceptive use, among married adolescent girls (15–19 years) in Bihar and Uttar Pradesh, India (N = 4893). **Table A5.** Fear of being called barren for descriptive purposes among married adolescent girls (15–19 years) in Bihar and Uttar Pradesh combined, India (N = 4893).

## Data Availability

Data are publicly available and can be accessed through the Harvard Dataverse public data portal (https://doi.org/10.7910/DVN/ZJPKW5).

## Abbreviations

AOR: Adjusted odds ratio
ARSH: Adolescent reproductive and sexual health
β Coef.: Beta coefficient
CI: Confidence interval
IUD: Intrauterine device
LAM: Lactational amenorrhea method
OBC: Other backward caste
OR: Odds ratio
SC: Scheduled caste
SD: Standard deviation
ST: Scheduled tribe
UDAYA: Understanding lives of adolescents and young adults
UP: Uttar Pradesh
VIF: Variance inflation factor
LMIC: Low-to middle-income countries
