# Supplementary material for: The association between early in marriage fertility pressure from in-laws’ and family planning behaviors, among married adolescent girls in Bihar and Uttar Pradesh, India
Source: Reprod Health. 2021 Mar 9;18:60. doi: 10.1186/s12978-021-01116-9 (PMC7941884; doi:10.1186/s12978-021-01116-9)
Supplement: Supplementary file 1 — Additional file 1: Table A1. Sociodemographic characteristics of the study sample among married adolescent girls (15–19 years) in Bihar and Uttar Pradesh, India (Bihar N = 3,182, UP N = 1711). Table A2. Pressure from in-laws’ to have child early in marriage by outcomes of ever contraception use, communication about number of children, time until first birth, and parity, among married adolescent girls (15–19 years) in Bihar and Uttar Pradesh state wise, India (Bihar N = 3182, and Uttar Pradesh N = 1711, 2202 for communication about number of children). Table A3. Unadjusted and adjusted logistic and linear regression between pressure from in-laws’ to have a child immediately after marriage by outcomes of ever contraception use, communication about number of children, time to birth, and parity, among married adolescent girls (15–19 years) in Bihar (N = 3182), and Uttar Pradesh (N = 1711), India. Table A4. Sensitivity analysis of unadjusted and adjusted multinomial regression between pressure from in-laws’ to have child early in marriage and ever modern contraceptive use, among married adolescent girls (15–19 years) in Bihar and Uttar Pradesh, India (N = 4893). Table A5. Fear of being called barren for descriptive purposes among married adolescent girls (15–19 years) in Bihar and Uttar Pradesh combined, India (N = 4893). [file 12978_2021_1116_MOESM1_ESM.docx]

**Appendix**

**Table A1:** Sociodemographic characteristics of the study sample among married adolescent girls (15-19 years) in Bihar and Uttar Pradesh, India (Bihar N=3,182, UP N=1,711).

|  | Bihar | | | UP | | |
| --- | --- | --- | --- | --- | --- | --- |
|  | **Overall N (%)** | **In-laws’ pressure** | | **Overall N (%)** | **In-laws’ pressure** | |
|  |  | **Yes, n (%)** | **No, n (%)** |  | **Yes, n (%)** | **No, n (%)** |
| Age (years) |  |  |  |  |  |  |
| 15 | 93 (1.83%) | 15 (1.74%) | 78 (3.19%) | 24 (1.24%) | 5 (2.11%) | 19 (1.09%) |
| 16 | 280 (9.69%) | 64 (8.20%) | 216 (10.18%) | 89 (4.68%) | 17 (8.11%) | 72 (4.09%) |
| 17 | 578 (18.37%) | 137 (18.54%) | 441 (18.32%) | 207 (13.40%) | 37 (15.96%) | 170 (12.96%) |
| 18 | 1,042 (31.97%) | 245 (33.48%) | 797 (31.48%) | 568 (32.89%) | 78 (31.42%) | 490 (33.14%) |
| 19 | 1,189 (37.14%) | 251 (38.04%) | 938 (36.84%) | 823 (47.78%) | 104 (42.40%) | 719 (48.71%) |
| Education (years), mean (SD) | 5.63 (0.18) | 5.49 (4.13) | 5.67 (4.43) | 6.70 (4.33) | 6.62 (4.61) | 6.71 (4.28) |
| Area of Residence |  |  |  |  |  |  |
| Rural | 1,871 (90.50%) | 455 (92.61%) | 1,416 (89.81%) | 1,142 (82.23%) | 167 (83.62%) | 975 (81.99%) |
| Urban | 1,311 (9.50%) | 257 (7.39%) | 1,054 (10.19%) | 569 (17.77%) | 74 (16.38%) | 495 (18.01%) |
| Religion |  |  |  |  |  |  |
| Hindu | 2,746 (87.87%) | 627 (88.99%) | 2,119 (87.50%) | 1,351 (77.97%) | 199 (81.59%) | 1,152 (77.35%) |
| Other Religions* | 436 (12.13%) | 85 (11.01%) | 351 (12.50%) | 360 (22.03%) | 42 (18.41%) | 318 (22.65%) |
| Caste |  |  |  |  |  |  |
| General | 256 (7.24%) | 658 (94.95%) | 2,268 (92.76%) | 252 (16.39%) | 205 (84.84%) | 1,254 (83.40%) |
| SC/ST/OBC** | 2,926 (92.76%) | 54 (5.05%) | 202 (7.96%) | 1,459 (83.61%) | 36 (15.16%) | 216 (16.60%) |
| Time since marriage (years), mean (SD) | 2.18 (1.55) | 2.15 (1.29) | 2.17 (1.32) | 2.16 (1.31) | 2.45 (1.49) | 2.09 (1.57) |
| Wealth quintile |  |  |  |  |  |  |
| Q1 (poorest) | 410 (13.86%) | 87 (12.69%) | 323 (14.24%) | 266 (13.75%) | 38 (13.84%) | 228 (13.73%) |
| Q2 (poorer) | 524 (19.04%) | 141 (20.78%) | 383 (18.47%) | 350 (21.54%) | 49 (23.21%) | 301 (21.25%) |
| Q3 (middle) | 655 (22.84%) | 147 (21.17%) | 508 (23.39%) | 414 (24.70%) | 69 (28.24%) | 345 (24.09%) |
| Q4 (richer) | 805 (26.02%) | 179 (29.83%) | 626 (24.77%) | 419 (23.97%) | 57 (20.61%) | 362 (24.55%) |
| Q5 (richest) | 788 (18.24%) | 158 (15.53%) | 630 (19.13%) | 262 (16.04%) | 28 (14.10%) | 234 (16.37%) |
| Total N | 3,182 (100%) | 712 (100%) | 2,470 (100%) | 1,711 (100%) | 241 (100%) | 1,470 (100%) |

*Other religions include Muslim, Christian, Buddhist, and Others.

** SC: Scheduled Caste, ST: Scheduled Tribe, OBC: Other Backward Caste

*Note:* Frequency and weighted proportions are reported for categorical variables. Weighted means and standard deviations are reported for continuous variables.

**Table A2:** Pressure from in-laws to have child early in marriage by outcomes of ever contraception use, communication about number of children, time until first birth, and parity, among married adolescent girls (15-19 years) in Bihar and Uttar Pradesh state wise, India (Bihar N=3,182, and Uttar Pradesh N=1,711, 2202 for communication about number of children).

|  | Overall, n (%) | Ever contraception use, n (%) | | Communication about number of children, n (%) | | Time until first birth (0-7 years), mean (SD) | Parity (range 0-4 births), mean (SD) |
| --- | --- | --- | --- | --- | --- | --- | --- |
| Pressure from in-laws’ | - | Yes | No | Yes | No/Don’t know |  |  |
| Bihar | | | | | | | |
| Yes | 712 (24.67%) | 70 (9.46%) | 642 (90.54%) | 439 (63.02%) | 273 (36.98%) | 1.73 (0.99) | 0.50 (0.50) |
| No | 2,470 (75.33%) | 300 (11.71%) | 2,170 (88.29%) | 1,306 (51.09%) | 1,164 (48.91%) | 1.61 (1.13) | 0.59 (0.69) |
| Total N | 3,182 (100%) | 370 (11.15%) | 2,812 (88.85%) | 1,745 (54.05%) | 1,437 (45.95%) | 1,593 | 3,128 |
| Uttar Pradesh | | | | | | | |
| Yes | 241 (14.64%) | 45 (15.80%) | 196 (84.20%) | 173 (70.55%) | 68 (29.45%) | 1.65 (1.07) | 0.34 (0.59) |
| No | 1,470 (85.36%) | 286 (18.08%) | 1,184 (81.92%) | 847 (57.36%) | 623 (42.64%) | 1.62 (0.92) | 0.41 (0.62) |
| Total N | 1,711 (100%) | 331 (17.74%) | 1,380 (82.26%) | 1,020 (59.30%) | 691 (40.70%) | 609 | 1,711 |

*Note:* Frequency and weighted proportions are reported for categorical variables. Weighted means and standard deviations are reported for continuous variables.

**Table A3:** Unadjusted and adjusted logistic and linear regression between pressure from in-laws to have a child immediately after marriage by outcomes of ever contraception use, communication about number of children, time to birth, and parity, among married adolescent girls (15-19 years) in **Bihar** (N=3,182), and **Uttar Pradesh** (N=1,711), India.

|  | Ever use of contraception | | Communication about number of children | | Time until birth (N=1593) | | Parity | |
| --- | --- | --- | --- | --- | --- | --- | --- | --- |
| In-laws’ pressure to have children | Unadjusted | Adjusted | Unadjusted | Adjusted | Unadjusted | Adjusted | Unadjusted | Adjusted |
|  | OR (95% CI) | AOR (95% CI) | OR (95% CI) | AOR (95% CI) | β Coef. (95% CI) | β Coef. (95% CI) | β Coef. (95% CI) | β Coef. (95% CI) |
| Bihar |  |  |  |  |  |  |  |  |
| No | ref | Ref | Ref | ref | ref | Ref | Ref | ref |
| Yes | 0.78 (0.51, 1.19) | 0.77 (0.51, 1.18) | 1.62 **(1.17, 2.26)** | 1.62 **(1.18, 2.22)** | 0.12 (-0.06, 0.29) | 0.3 (-0.11, 0.17) | -0.09 **(-0.17, -0.001)** | -0.16 **(-0.27, -0.06)** |
| Uttar Pradesh |  |  |  |  |  |  |  |  |
| No | ref | Ref | Ref | ref | ref | Ref | ref | ref |
| Yes | 0.88 (0.52, 1.50) | 0.90 (0.52, 1.56) | 1.77 **(1.23, 2.54)** | 1.85 **(1.29, 2.65)** | 0.03 (-0.27, 0.34) | -0.13 (-0.43, 0.16) | -0.07 (-0.17, 0.30) | -0.4 (-0.11, 0.30) |

*Note:* Adjusted for age, education, residence, religion, caste, time since marriage, wealth quintiles for Bihar. OR – odds ratio, CI – confidence interval, ref – reference.

**Table A4:** Sensitivity analysis of unadjusted and adjusted multinomial regression between pressure from in-laws to have child early in marriage and ever modern contraceptive use, among married adolescent girls (15-19 years) in **Bihar** **and Uttar Pradesh**, India (N=4,893).

|  | Ever modern use of contraception | |
| --- | --- | --- |
| Variable | Unadjusted | Adjusted |
| In-laws’ pressure to have children | RRR (95% CI) | ARRR (95% CI) |
| Traditional method |  |  |
| No | ref | ref |
| Yes | 0. 69 (0.41, 1.17) | 0.66 (0.40, 1.10) |
| Modern method |  |  |
| No | ref | ref |
| Yes | 0.80 (0.52, 1.25) | 0.94 (0.60, 1.46) |

**Table A5:** Fear of being called barren for descriptive purposes among married adolescent girls (15-19 years) in Bihar and Uttar Pradesh combined, India (N=4,893).

| Fear of being called barren by in-laws’ and others | Overall, n (%) | Ever contraception use, n (%) | | Communication about number of children, n (%) | | Time until first birth (0-7 years), mean (SD) | Parity (range 0-4 births), mean (SD) |
| --- | --- | --- | --- | --- | --- | --- | --- |
|  |  | Yes | No | Yes | No/Don’t know |  |  |
| No/Can’t say | 3,920 (81.17%) | 605 (16.67%) | 3,315 (83.33%) | 2,142 (55.10%) | 1,778 (44.90%) | 1.60 (0.98) | 0.50 (0.64) |
| Yes | 973 (18.83%) | 96 (9.32%) | 877 (90.68%) | 623 (67.02%) | 350 (32.98%) | 1.80 (1.18) | 0.44 (0.64) |
| Total | 4,893 (100%) | 701 (84.71%) | 4,192 (15.29%) | 973 (18.83%) | 3,920 (81.17%) | 2,202 | 4,893 |

*Note:* Frequency and weighted proportions are reported for categorical variables. Weighted means and standard deviations are reported for continuous variables.
